# Targeting Of Somatic Hypermutation By *immunoglobulin* Enhancer And Enhancer-Like Sequences

**DOI:** 10.1371/journal.pbio.1001831

**Published:** 2014-04-01

**Authors:** Jean-Marie Buerstedde, Jukka Alinikula, Hiroshi Arakawa, Jessica J. McDonald, David G. Schatz

**Affiliations:** 1 Department of Immunobiology, Yale University School of Medicine, New Haven, Connecticut, United States of America; 2 Howard Hughes Medical Institute, Yale University School of Medicine, New Haven, Connecticut, United States of America; 3 IFOM-FIRC Institute of Molecular Oncology Foundation, Milano, Italy; Scripps Research Institute, United States of America

## Abstract

Somatic hypermutation (SH) generates point mutations within rearranged *immunoglobulin* (*Ig*) genes of activated B cells, providing genetic diversity for the affinity maturation of antibodies. SH requires the activation-induced cytidine deaminase (AID) protein and transcription of the mutation target sequence, but how the *Ig* gene specificity of mutations is achieved has remained elusive. We show here using a sensitive and carefully controlled assay that the *Ig* enhancers strongly activate SH in neighboring genes even though their stimulation of transcription is negligible. Mutations in certain E-box, NFκB, MEF2, or Ets family binding sites—known to be important for the transcriptional role of *Ig* enhancers—impair or abolish the activity. Full activation of SH typically requires a combination of multiple *Ig* enhancer and enhancer-like elements. The mechanism is evolutionarily conserved, as mammalian *Ig lambda* and *Ig heavy chain* intron enhancers efficiently stimulate hypermutation in chicken cells. Our results demonstrate a novel regulatory function for *Ig* enhancers, indicating that they either recruit AID or alter the accessibility of the nearby transcription units.

## Abstract 2

*Immunoglobulin* gene enhancers have a conserved function in targeting somatic hypermutation to *immunoglobulin* genes, thereby supporting the production of high affinity antibodies.

## Author Summary

During the B cell immune response, *immunoglobulin* (*Ig*) genes are subject to a unique mutation process known as somatic hypermutation that allows the immune system to generate high-affinity antibodies. Somatic hypermutation preferentially affects *Ig* genes, relative to other genes, and this is important in preventing catastrophic levels of general genomic mutations that could lead to B cell cancers. We hypothesized that this preferential targeting of somatic hypermutation is assisted by specific DNA sequences in or near *Ig* genes that focus the action of the mutation machinery on those genes. In this study, we show that *Ig* genes across species—from human, mouse, and chicken—do indeed contain such mutation targeting sequences and that they coincide with transcriptional regulatory regions known as enhancers. We show that combinations of *Ig* enhancers cooperate to achieve strong mutation targeting and that this action depends on well-known transcription factor binding sites in these enhancer elements. Our findings establish an evolutionarily conserved function for enhancers in somatic hypermutation targeting, which operates by a mechanism distinct from the conventional enhancer function of increasing levels of transcription. We propose that combinations of *Ig* enhancers target somatic mutation to *Ig* genes by recruiting the mutation machinery and/or by making the *Ig* genes better substrates for mutation.

## Introduction

The appearance of point mutations within the rearranged *immunoglobulin* (*Ig*) genes of B cells, which leads eventually to the selection and production of high-affinity antibodies, is called somatic hypermutation (SH) [1],[2]. SH requires transcription of the *Ig* genes [3] and expression of the activation-induced cytidine deaminase (AID) protein encoded by the *AICDA* gene [4],[5]. AID is believed to initiate all three types of B cell–specific *Ig* gene diversification—SH, *Ig* gene conversion (GCV), and *Ig* class switch recombination—by deaminating cytidines within the *Ig* loci [6]–[8].

While many non-*Ig* genes accrue mutations in AID-expressing B cells as a result of SH, *Ig* genes mutate at levels that are typically several orders of magnitude greater than those of non-*Ig* genes [9]–[12]. The question of how SH is preferentially targeted to *Ig* loci has been studied and debated for over 20 years. Pioneering experiments using chimeric gene constructs in transgenic mice indicated that sequences overlapping with the *Ig light chain* and *Ig heavy chain* enhancers distinguish the *Ig* genes as mutation targets [13]–[15]. Other early transgene studies indicated that Ig V region sequences themselves are not required for SH [16] and that active heterologous promoters can support SH [13],[17]. However, further insight into the nature of the putative *cis*-acting regulatory elements was hampered by the laborious transgene experimental system, the relatively low mutation rates of the chimeric genes, and the fluctuation of mutation rates among transgenic lines, perhaps due to integration site effects and copy number variations. A further problem arose from the fact that the putative hypermutation-stimulating sequences included the known enhancers, making it difficult to differentiate between the effects of these sequences on transgene hypermutation versus transgene transcription (reviewed in [18]).

The hypothesis that SH is targeted preferentially to *Ig* genes by the *Ig* enhancers was subsequently called into question when germline deletions of individual murine *Ig* enhancers—the same sequences previously implicated in the hypermutation of chimeric transgenes—did not abolish SH within the respective loci [19]–[21]. It also became apparent that expression of either AID or the related cytidine deaminases APOBEC-3A or APOBEC-3B increased mutation frequencies in the genomes of fibroblasts [22], *Escherichia coli* [23], yeast [24], and human breast cancer cells [25]. These findings and others (reviewed in [9],[18]) raised widespread doubts about the relevance of specific *cis*-acting SH targeting elements in *Ig* loci. In particular, *Ig* enhancers were no longer regarded as likely SH targeting elements, and it was increasingly felt that they increased SH solely by increasing *Ig* gene transcription. Attention has recently focused on RNA polymerase II (Pol II)–associated factors that interact with AID and play roles in transcriptional stalling [26] and RNA processing [27], processes that are likely to be critical for generating the single strand DNA substrate required by AID (reviewed in [9],[28]). However, these broadly acting factors do not provide a ready explanation for the strong preference that SH exhibits for *Ig* genes over non-*Ig* genes. Consequently, this has remained a central unresolved issue in the field.

The chicken B cell line DT40, whose genome is easily modified by targeted gene integration [29], is a powerful model to investigate AID-mediated gene diversification [30]. DT40 variegates its rearranged *Ig light chain (cIgλ*) gene primarily by GCV [31], but diversification occurs by SH if either upstream GCV donor sequences or uracil DNA glycosylase (UNG) are missing [7],[32]. Evidence for the stimulation of *cIgλ* GCV by *cis*-acting sequences in DT40 has been detected by the analysis of endogenous *cIgλ* gene diversification [33], transgene GCV [34], and transgene hypermutation [35]. Reminiscent of the early experiments in transgenic mice, SH of a *green fluorescent protein* (*GFP*) knock-in transgene in DT40 cells depended on the nearby presence of a 10-kb fragment of the *cIgλ* locus, which was named *diversification activator* (*DIVAC*) [35]. Deletion analysis of *DIVAC* led to the identification of two core regions downstream of the *cIgλ C-region* that cooperate with each other and with other parts of the 10-kb sequence to stimulate SH of the adjacent *GFP* transcription unit [36]. However, a clearer definition of the DIVAC code proved challenging using the original GFP assay because of functional redundancy within the 10-kb sequence and difficulty in measuring the DIVAC activity of elements shorter than 500 bp [35]–[37]. Furthermore, murine *Ig lambda* (*Igλ*) and *Ig kappa* (*Igκ*) enhancer sequences displayed disappointingly low DIVAC activity in DT40 cells [36],[38]. Hence, the identity of key SH targeting sequences and the extent to which these sequences have been conserved during vertebrate evolution have remained undetermined.

We have now developed a highly sensitive assay that allows analysis of the SH targeting activity of small DNA elements, largely overcoming the shortcomings of previous experimental strategies. Using this new assay, we demonstrate that chicken, mouse, and human *Ig* locus enhancers and enhancer-like elements are core DIVAC sequences that work together to target SH. Regardless of which species they derive from, these elements rely for function on a common set of well-characterized transcription factor binding motifs, highlighting the evolutionary conservation of the SH targeting mechanism. These findings are likely to have implications for the mistargeting of SH to non-*Ig* genes and the origins of B cell lymphoma.

## Results

### A Highly Sensitive DIVAC Assay

**Figure 1:**
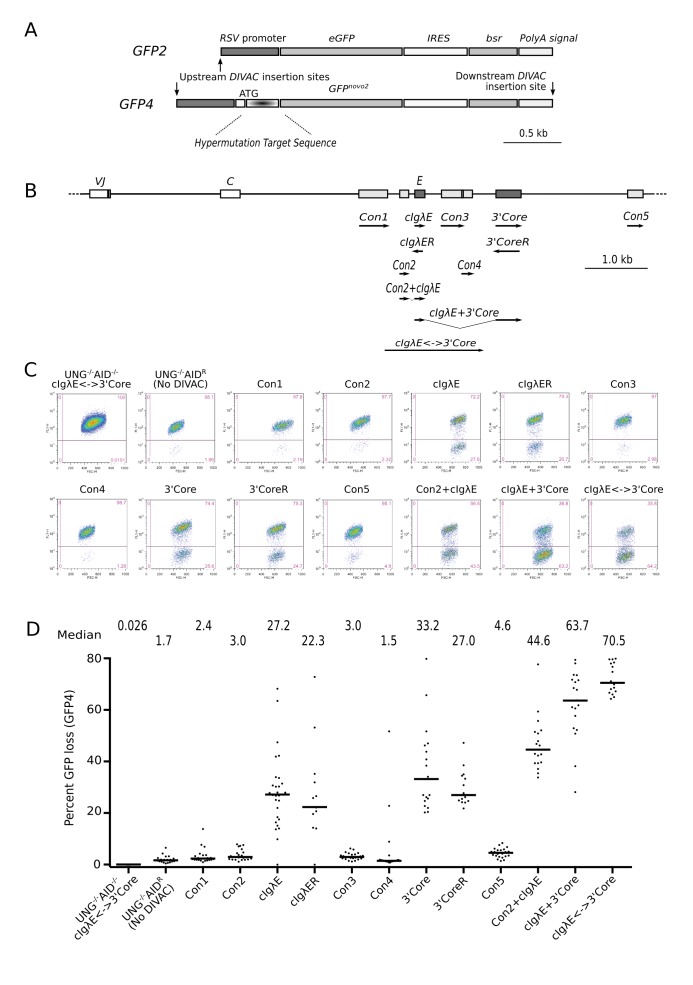
GFP4 assay detects stimulation of hypermutation by short conserved fragments of the chicken *Igλ* locus. (A) Diagram of the *GFP2* and *GFP4* hypermutation reporters. RSV, rous sarcoma virus; bsr, blasticidin resistance gene; IRES, internal ribosome entry site; ATG, the start codon of the hypermutation target sequence–GFP fusion protein encoded by *GFP4*. (B) Map of the rearranged chicken *Igλ* (*cIgλ*) locus with the location of sequences conserved among avian species indicated by rectangles. Sequences tested are shown below, with their orientation relative to *GFP4* indicated by arrows. VJ, rearranged variable region gene; C, constant region; E, enhancer. (C) Flow cytometry profiles of representative subclones of primary transfectants carrying either *GFP4* alone (UNG^−/−^AID^R^) or *GFP4* combined with the sequence specified above each plot. All transfectants are UNG-deficient, AID-reconstituted, except UNG^−/−^AID^−^cIgλE↔3′Core, which does not express AID. (D) Graph showing the percent GFP loss of individual subclones. Each dot represents a subclone. The median GFP loss for each group of subclones is indicated by the bar and numerically displayed above the graph.

We previously developed an assay for DIVAC function that made use of a reporter cassette, termed *GFP2*, consisting of a strong viral promoter driving expression of *GFP* and a drug resistance gene (Figure 1A) [35]. In this assay, *GFP2*, with or without a flanking test sequence, was inserted by homologous recombination into the DT40 genome, and GFP expression was monitored in subclones by flow cytometry. Loss of GFP expression was entirely dependent on AID, was due to point mutations in *GFP*, and could be stimulated more than 100-fold by the presence of a strong *DIVAC* element adjacent to the *GFP2* cassette [35]. Importantly, three previous studies demonstrated that DIVAC-dependent stimulation of *GFP* mutation was not accompanied by substantial changes in *GFP* transcription as measured by several methods, demonstrating that DIVAC stimulates SH by a mechanism independent of an increase in transcription [35]–[37].

To increase the sensitivity of the DIVAC assay, we modified the *GFP2* reporter by the insertion of a 5′ untranslated sequence upstream of the methionine start codon and a hypermutation target sequence between the start codon and the *GFP* open reading frame, yielding the new reporter *GFP4* (Figure 1A). The 249-bp hypermutation target sequence consists of repetitions of TGG, CAA, and CAG codons frequently positioned in the context of SH hotspot motifs WRCY/RGYW (W = A or T; R = A or G; Y = C or T). Transition mutations at the second or third position of the TGG codons or at the first position of the CAA and CAG codons will introduce nonsense mutations, precluding the translation of the *GFP* open reading frame (Figure S1).

To further increase the frequency at which mutations and stop codons are generated, the GFP4 assay is performed in *UNG*-deficient cells, which accumulate exclusively C-to-T and G-to-A transition mutations and display a 7-fold increased rate of SH [32], most likely because AID-induced uracils cannot be excised and repaired before replication. To assay *DIVAC-GFP4* combinations at a defined chromosomal position, we generated a recipient cell line, UNG^−/−^AID^R/puro^, in which (i) both endogenous *UNG* genes were disrupted and the coding sequences of both endogenous *AICDA* genes were deleted, (ii) AID expression was reconstituted by inserting an *AICDA* cDNA expression cassette under the influence of the β-*actin* promoter into one *AICDA* locus, and (iii) the position of the second *AICDA* locus was marked by a puromycin resistance gene. When this cell line is transfected by *AICDA* locus–targeting constructs containing *DIVAC-GFP4*, targeted integrants into the marked *AICDA* locus are easily identified by the loss of puromycin resistance.

### Conservation of *DIVAC* Sequences

Alignment of the *cIgλ* locus with the corresponding sequence of turkey, zebra finch, and ground finch revealed seven evolutionarily conserved sequence contigs downstream of the *C*-*region* (Figures 1B and S2). Two of these corresponded closely to regions we had previously demonstrated to be important for DIVAC function in the context of larger DNA elements [36]: the *cIgλ* enhancer (*cIgλE*) [39] and the *3′Core*. The conserved sequence regions were cloned into the upstream *DIVAC* insertion site of *GFP4* (the default site used in all experiments except where indicated) and transfected into UNG^−/−^AID^R/puro^ cells. Primary transfectants with targeted integration of a construct were subcloned, and 24 subclones were analyzed for GFP loss by flow cytometry 12 d after subcloning (Figure 1C and 1D). Transfectants containing *cIgλE* or *3′Core*, in either orientation (reverse orientation indicated by “R”), showed median GFP loss levels of 20%–30%, whereas levels of GFP loss in transfectants of the other conserved sequences (*Con1*–*Con5*) were close to the 1.7% median value observed in the no DIVAC control transfectant, UNG^−/−^AID^R^. Interestingly, the *Con2* sequence, which displayed activity close to background on its own, substantially increased GFP loss when combined with *cIgλE* in Con2+cIgλE cells (44.6%). The highest levels of GFP loss were seen when *cIgλE* and the *3′Core* were combined (63.7%) or when they were tested together with their intervening sequence (cIgλE↔3′Core; 70.5%). Importantly, GFP loss in UNG^−/−^AID^−/−^cIgλE↔3′Core cells (lacking the *AICDA* expression cassette) was almost 3,000-fold lower than in cIgλE↔3′Core cells and about 60-fold lower than in UNG^−/−^AID^R^ cells.

These results illustrate several points. First, the DIVAC-GFP4 assay is capable of detecting robust stimulation of SH by short DNA fragments, which heretofore has not been possible. Second, these results directly confirm the role *of cIgλE* and *3′Core* as core *DIVAC* elements [36]. Third, in the absence of *DIVAC*, GFP loss from *GFP4* in *UNG^−/−^* cells is 15- to 20-fold higher than we detect with *GFP2* in wild-type cells (see below, and [35],[36]), likely reflecting both the increased sensitivity of *GFP4* and an increase of *DIVAC*-independent mutations in the *UNG*-deficient background. Finally, in the absence of AID, *UNG* deficiency does not lead to substantial GFP loss, even in the presence of a strong *DIVAC* element. Hence, despite the repair-deficient context, both *DIVAC*-dependent and *DIVAC*-independent GFP loss in the GFP4 assay require AID.

Sequencing of the hypermutation target region amplified from cIgλE↔3′Core cells 6 wk after subcloning revealed frequent transition mutations at G/C bases with a hotspot preference as expected for SH in *UNG*-deficient DT40 cells (Figure S1). Many of these mutations yielded stop codons, explaining the efficient GFP loss seen in cIgλE↔3′Core cells.

### *DIVAC* Elements Require Transcription Factor Binding Motifs

**Figure 2:**
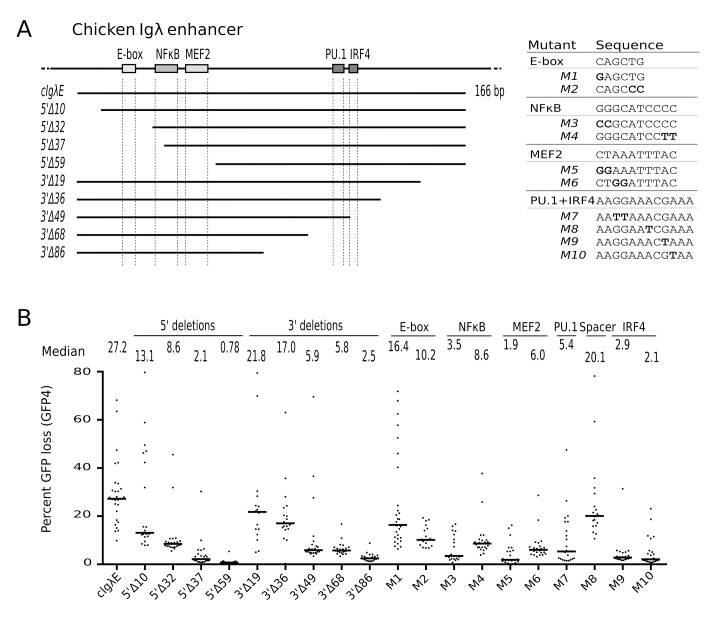
Deletion and mutation analysis of the chicken *Igλ* enhancer. (A) Diagram of the *cIgλE* fragment with truncations indicated below and conserved transcription factor binding motifs depicted as rectangles. The sequences of binding sites and binding site mutants are shown on the right. (B) GFP loss of subclones in the presence of full-length, truncated, and mutated *cIgλE* sequences.

*cIgλE* includes an E-box as well as NFκB (nuclear factor kappa B), MEF2 (myocyte-specific enhancer factor 2), and PU.1-IRF4 (interferon regulatory family-4) binding motifs, all of which are remarkably conserved among avian species (Figure S2B). Deletions starting either from the 5′ or the 3′ end of *cIgλE* progressively decreased GFP loss in the DIVAC assay (Figure 2A and 2B). Once the 5′ deletions reached the NFκB motif (5′Δ37), GFP loss fell to background levels. Similarly, 3′ end deletions including the IRF4 motif in 3′Δ49 cells strongly reduced GFP loss.

The role of specific binding site motifs was further investigated by mutation of consensus residues in these sites (Figure 2A and 2C). Whereas mutations in the NFκB, MEF2, PU.1, or IRF4 motifs strongly decreased GFP loss, mutations in the E-box caused a more modest reduction, and a mutation in the spacer between the PU.1 and IRF4 motifs was well tolerated (Figure 2C). These results indicate that *cIgλE* requires the integrity of multiple transcription factor binding sites in its 5′ and 3′ halves for full activity.

Little was known about *3′Core*, the second autonomous *DIVAC* sequence of the chicken *Igλ* locus. Deletion of the first 42 and the last 99 bp did not affect GFP loss (5′Δ42_3′Δ99), whereas many deletions in the central part of the fragment reduced GFP loss (Figure S3A and S3B). Search algorithms for transcription factor binding motifs predicted, among others, six evolutionarily conserved binding motifs in the parts of *3′Core* where deletions compromised activity: three E-boxes and three other putative sites, referred to as pCBF (core binding factor), pC/EBP (CCAAT enhancer binding protein), and pPU.1 (Figure S2C) (where “p” designates a putative binding site for which experimental evidence linking it to the factor is lacking). Deletion or mutation of any one of these motifs, with the exception of pPU.1, reduced GFP loss substantially, with the strongest effects seen for E-box2, pCBF, and pC/EBP, which lie close together in the central part of the fragment (Figure S3C and S3D). Thus, evolutionarily conserved transcription factor binding motifs are also critical for the *DIVAC* function of *3′Core*. We note that many more sites were predicted in silico than were tested, and the factors that might bind to these and the tested sites, particularly pCBF and pC/EBP, remain unknown.

### The Human *Igλ* Enhancer as a Surprisingly Strong *DIVAC*

Alignment of human, murine, and chicken *Igλ* enhancer sequences revealed striking conservation of the E-box and NFκB, MEF2, PU.1, and IRF4 binding motifs [40],[41], while the mammalian sequences possess an additional E-box about 50 bp downstream of the PU.1 site (Figure S4A). Since the conserved transcription factor binding motifs were important for the DIVAC function of *cIgλE*, we reasoned that the mammalian enhancers might also be active DIVAC elements despite low sequence conservation of the intervening sequences.

**Figure 3:**
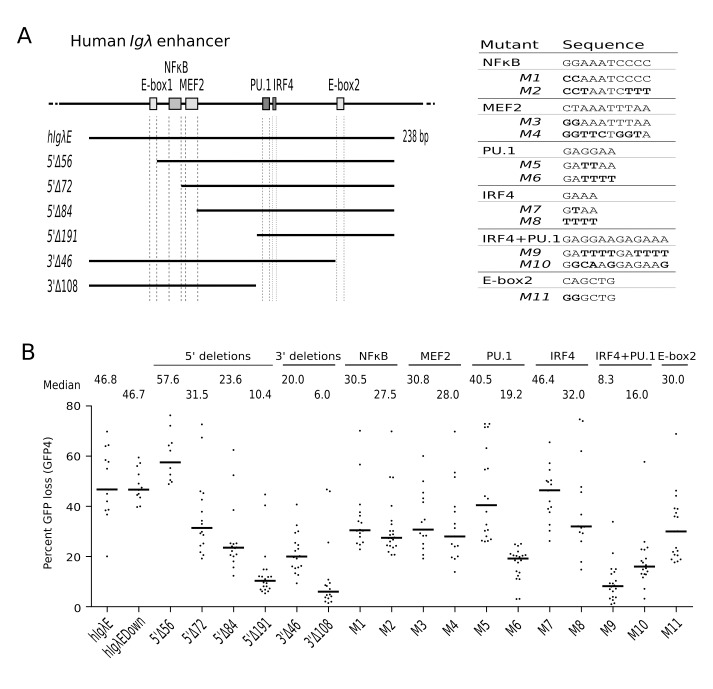
Efficient stimulation of hypermutation by the human *Igλ* enhancer. (A) Diagram of the *hIgλE* fragment with truncations indicated below and conserved transcription factor binding sites depicted as rectangles. The sequences of binding sites and binding site mutants are shown on the right. (B) GFP loss of subclones in the presence of full-length, truncated, and mutated *hIgλE*. hIgλEDown cells carry *hIgλE* downstream of *GFP4*.

We began by testing the human *Igλ* enhancer (*hIgλE*) in either the upstream or downstream insertion site of *GFP4* (Figure 1A), which yielded a remarkable 46% GFP loss (Figure 3B), almost twice the activity of *cIgλE* (27.2%). Removal of the upstream E-box in 5′Δ56 did not decrease DIVAC activity, whereas larger 5′ deletions reduced activity (Figure 3A and 3B). However, even after removal of the upstream E-box, NFκB, and MEF2 sites, the 5′Δ84 fragment was still capable of supporting 23.6% GFP loss, almost as high as the activity of full-length *cIgλE* and much higher than the activity of the comparable deletion fragment (5′Δ59) of *cIgλE* (Figure 2B). These results suggest that the 3′ portion of *hIgλE* contains important elements and that the downstream E-box might compensate for loss of the upstream E-box-NFκB-MEF2 sites. Consistent with this, a 3′ deletion including the downstream E-box (3′Δ46) reduced GFP loss to 20%—roughly the activity of full-length *cIgλE*—and a larger 3′ deletion removing the composite PU.1-IRF4 site (3′Δ108) strongly reduced GFP loss to 6% (Figure 3B), similar to the low activity of the comparable *cIgλE* 3′Δ68 fragment (Figure 2B). In the strongly active *hIgλE*, point mutations in individual motifs reduced activity, although typically less than 2-fold, and only mutation of both components of the composite PU.1-IRF4 site had a strong effect on activity (Figure 3A and 3B). Therefore, *hIgλE* is both more active and apparently more robust than *cIgλE*, being less sensitive to mutation of individual motifs. The major difference between the human and chicken enhancers appears to lie in sequences in their 3′ portions.

These results demonstrate, to our knowledge for the first time, a substantial conservation of DIVAC function from human to chicken sequences. They also reveal parallels between the enhancement of SH and the enhancement of transcription by the *Igλ* enhancer because the transcription factor binding sites long known to be important for the regulation of *Igλ* transcription [41]–[43] are also critical for DIVAC function.

### Enhancers as DIVAC Elements in Mammalian *Igh* and *Igκ* Loci

**Figure 4:**
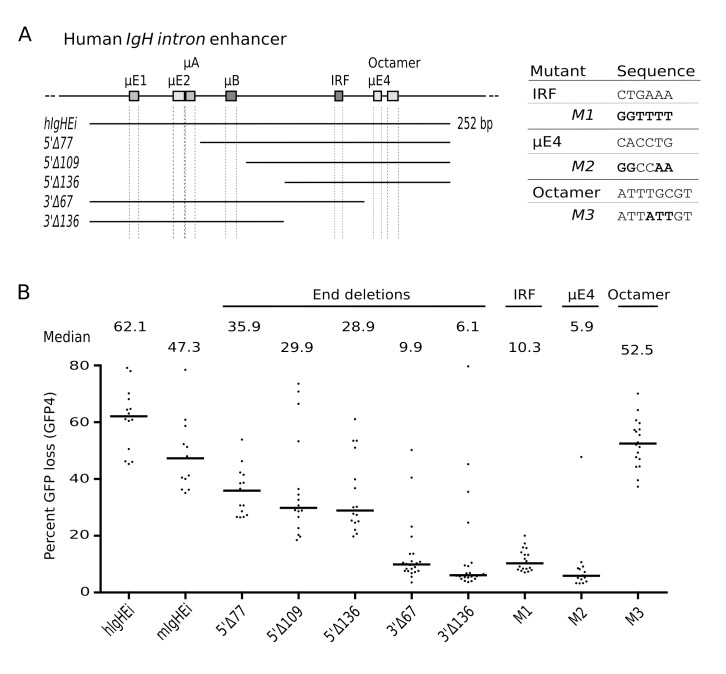
Important *DIVAC* motifs map to the 3′ part of the human *IgH* intron enhancer. (A) Diagram of the *hIgHEi* fragment with truncations indicated below and transcription factor binding sites depicted as rectangles. The sequences of binding sites and binding site mutants are shown on the right. (B) GFP loss in the presence of *hIgHEi*, *mIgHEi*, or truncated or mutated versions of *hIgHEi*.

Sequence homologues of mammalian *Ig heavy chain* intron enhancers (*IgHEi*) could not be identified in birds, and an enhancer in the intron between the duck *Jμ* and *Cμ* segments showed no obvious conservation with mammalian counterparts apart from the presence of multiple E-boxes [44]. Human (*hIgHEi*) and murine (*mIgHEi*) enhancer fragments contain conserved YY1 (yin yang 1) (μE1), E-box (μE2 and μE4), Ets1 (μA), PU.1 (μB), IRF, and Octamer transcription factor binding sites, and less well conserved regions μE5 and μE3 [45],[46] (Figure 4A and S4B). Since these sites overlap substantially with those important for DIVAC function in *cIgλE*, *3′Core*, and *hIgλE*, we reasoned that the mammalian *IgHEi* elements might also have SH targeting activity. Strikingly, *hIgHEi* and *mIgHEi* yielded high levels of GFP loss (62.1% and 47.3%, respectively; Figure 4B), well above that of *cIgλE* and *3′Core*, and similar to that observed with *hIgλE*.

To investigate the role of the well-known binding sites, *hIgHEi* was subject to deletion and mutation analysis. Whereas a 5′ deletion of *hIgHEi* including the μE1, μE2, μA, and μB sites only moderately decreased GFP loss in 5′Δ109 and 5′Δ136 cells, 3′ deletions including the Octamer, μE4, and IRF sites strongly decreased GFP loss in 3′Δ67 and 3′Δ136 cells. Consistent with the importance of the 3′ part of *hIgHEi*, mutations of either the μE4 or IRF site strongly decreased GFP loss, whereas an Octamer site mutation had little effect. Thus, the binding sites in the 5′ portion, although able to boost activity of the 3′ portion, are unable to compensate for loss of the IRF or μE4 sites in the 3′ portion. We conclude that mammalian *IgHEi* sequences are potent DIVAC elements in chicken cells.

**Figure 5:**
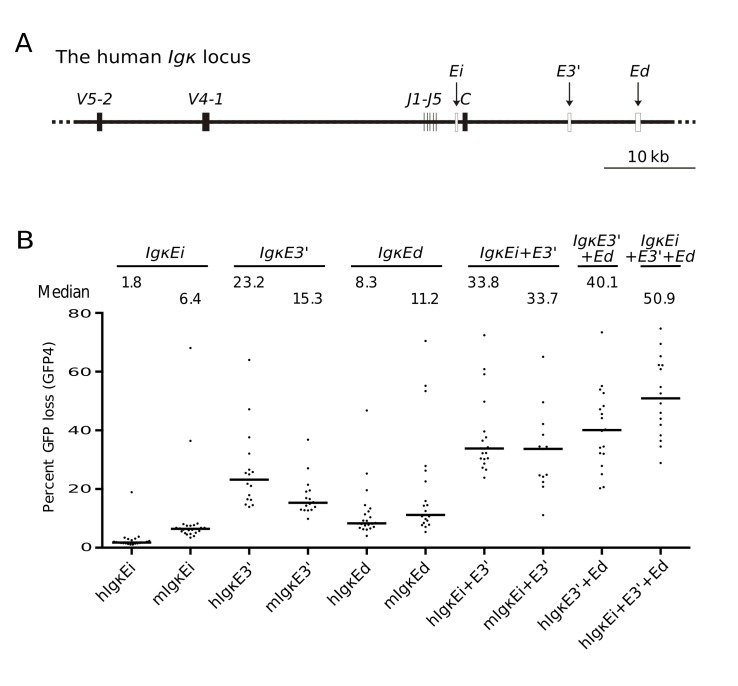
*Igκ* enhancers synergistically activate hypermutation. (A) Map of the human *Igκ* locus showing the locations of the three *Igκ* enhancers as open rectangles. V, variable gene segment; J, joining gene segment; C, constant region. (B) GFP loss in the presence of individual human and murine *Igκ* enhancers and enhancer combinations.

Homologues of the mammalian *Ig kappa chain* (*Igκ*) enhancers are also not present in avian species, which contain only a single *Igλ light chain* locus. The three *Igκ* enhancers, intron (*IgκEi*), 3′ (*IgκE3′*), and Ed (*IgκEd*) [47], of mice and humans (Figures 5A and S5) induced low or modest levels of GFP loss when assayed on their own (Figure 5B), consistent with previous analyses [36],[38]. However, when two *Igκ* enhancers were combined (*IgκEi*+*IgκE3′* or *IgκE3′*+*IgκEd*), GFP loss markedly increased, and when the three human *Igκ* enhancers were combined, GFP loss reached 50.9% (Figure 5B). This shows that the known synergy of the *Igκ* enhancers with respect to the activation of *Igκ* transcription ([47] and references therein) also holds true for their DIVAC function, even in an avian B cell line lacking an endogenous *Igκ* locus.

### Congruence between the GFP4 and GFP2 Assays

To confirm our results in a repair-proficient cellular context (UNG-proficient DT40 cells) and in a different genomic integration site (the deleted rearranged *Igλ* locus), we tested various *cIgλ DIVAC* elements using the GFP2 assay. The full *cIgλ DIVAC* region (the 9.8-kb *W* fragment that includes the rearranged VJλ region and all downstream *cIgλ* sequences) yielded about 10% GFP loss using *GFP2* (Figure S6B and S6C), consistent with our previous study [35]. In general, the rank order of activities of *DIVAC* elements was similar between the GFP2 and GFP4 assays (compare Figures S6C and 1D). Comparison of median GFP loss levels indicated that the GFP2 assay is approximately 20–50 times less sensitive than the GFP4 assay (e.g., for *cIgλE* and *3′Core*, respectively: 27.2% and 33.2% median GFP loss with *GFP4*, and 0.54% and 0.75% median GFP loss with *GFP2*). However, with the *cIgλE*↔*3′Core* fragment, GFP loss in the GFP2 assay (6.7%) was only about 10-fold lower than in the GFP4 assay (70.5%), probably because of saturation of the GFP4 assay in the presence of this highly active *DIVAC* element (see Protocol S1). We also used the GFP2 assay to confirm that *Con2* (which lacks activity on its own) was able to substantially boost the activity of *cIgλE* (Figure S6C). A limited deletion and mutation analysis of *Con2* (Figure S6A) using the GFP2 assay (Figure S6C) and the GFP4 assay (Figure S6D) demonstrated that functional cooperation between *Con2* and *cIgλE* required only the 3′ portion of *Con2* and was dependent on one of the two putative IRF binding motifs (pIRF-down) in this region. We conclude that there is good congruence between the results of the GFP4 and GFP2 assays and that the less sensitive GFP2 assay is preferable for analysis of highly active *DIVAC* elements.

### Newly Identified Shadow Enhancers Act as Strong *DIVAC* Elements in the Murine *Igλ* Locus

**Figure 6:**
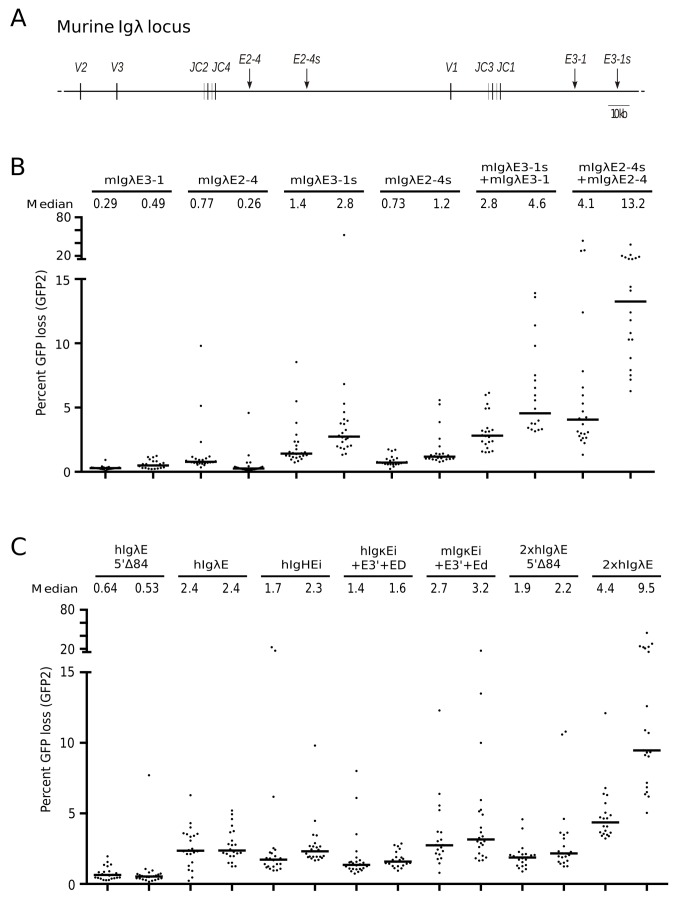
Murine *Igλ* shadow enhancers are strong *DIVACs* and synergize with the canonical enhancers. (A) Map of the murine *Igλ* locus showing the locations of the known and newly discovered shadow enhancers (arrows). V, variable gene segment; J, joining gene segment; C, constant region. (B) GFP loss in the presence of individual murine *Igλ* enhancers and canonical enhancer–shadow enhancer combinations (GFP2 assay). (C) GFP loss in the presence of mammalian enhancers as single copy, multimers, or combinations (GFP2 assay). In (B) and (C), subclones from two independent primary transfectants for each construct were analyzed, as indicated above each plot.

The murine *Igλ* locus contains two enhancers, *mIgλE3-1* and *mIgλE2-4*, due to a duplication of a pair of J-C regions and their downstream enhancer (Figure 6A) [48]. These enhancers are relatively weak *DIVAC* elements on their own (0.4%–0.5% GFP loss in the GFP2 assay; Figure 6B), consistent with our previous analysis [36]. This suggested the need for other sequences in the locus to cooperate with *mIgλE3-1* and *mIgλE2-4* to support efficient SH of murine *Igλ* (note that cooperation between *mIgλE3-1* and *mIgλE2-4* is not possible in some rearranged *Igλ* loci because rearrangement of upstream V2 or V3 gene segments to the JC3 or JC1 clusters deletes *mIgλE2-4*). However, the identity of such putative cooperating elements was unclear because other murine *Igλ* enhancers were not known.

Intriguingly, BLAST searches revealed the presence of *IgλE* homologues 20–25 kb downstream of *mIgλE3-1* and *mIgλE2-4* (Figure 6A), which we refer to as *mIgλE3-1s* and *mIgλE2-4s* because of their resemblance to shadow enhancers [49]. The newly identified elements are 95% identical to one another and about 70% identical to the canonical enhancers, with the conservation including many of the transcription factor binding motifs shown to be important for DIVAC function of the chicken and human *Igλ* enhancers (Figure S4A). When tested for DIVAC function, *mIgλE3-1s* and *mIgλE2-4s* were substantially more active than the canonical enhancers in both the GFP2 (Figure 6B) and GFP4 assays (data not shown). Strikingly, the combination of a shadow enhancer with its neighboring canonical enhancer induced GFP loss strongly and synergistically (Figure 6B), in the case of *mIgλE2-4* plus *mIgλE2-4s* to levels almost as high as that seen for the entire *cIgλ W* fragment. These results reveal that strong SH targeting elements can be constructed from combinations of enhancers and enhancer-like elements in the murine *Igλ* locus, as is true also for chicken *Igλ*. Furthermore, they demonstrate our ability to identify strong *DIVAC* elements in the murine *Igλ* locus on the assumption that *Igλ* enhancer-like sequences activate SH.

We extended this by investigating the activity of other combinations of elements, continuing to use the GFP2 assay. Consistent with the GFP4 data, *hIgλE*, *hIgHEi*, and the combined murine *Igκ* enhancers supported levels of GFP loss that were more than 20-fold above the background of AID^R^ cells (0.1%), whereas the 5′Δ84 deletion mutant of *hIgλE* was less active (Figure 6C). Duplication of the truncated *5′*Δ84 or the full-length *hIgλE* increased levels of GFP loss from about 0.6% and 2.4% to about 2.0% and 6%, respectively, showing that even the interaction between identical sequences can lead to a synergistic increase of DIVAC function, similar to the well-known effects of multimerization of enhancer sequences on transcriptional activity [50].

### *DIVAC* Elements Have Little Effect on Transcription of *GFP4*

**Figure 7:**
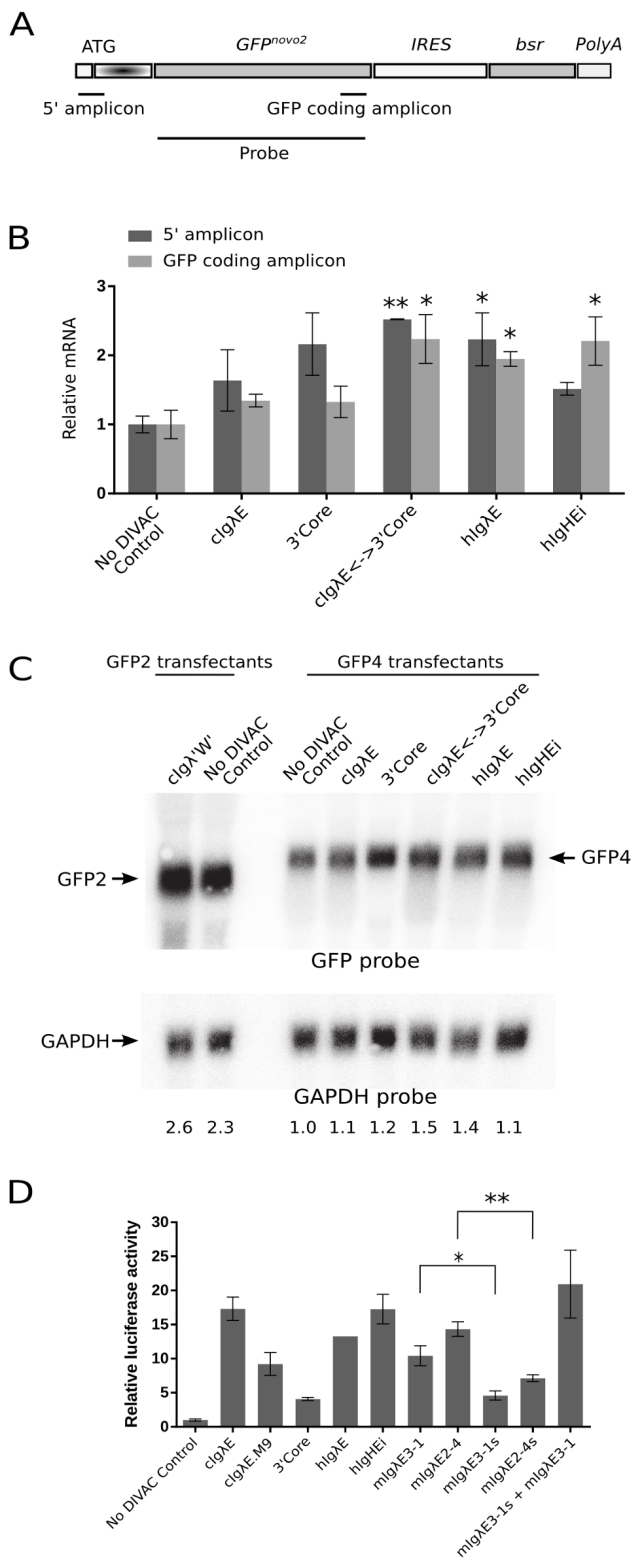
Analysis of parameters of transcription for *DIVAC* elements. (A) Diagram of *GFP4* (the RSV promoter is not shown) showing location of amplicons for the reverse transcription quantitative PCR analyses and the GFP probe used for the Northern blot. (B) Relative GFP mRNA levels in *GFP4* transfectants as determined by quantitative PCR. GFP transcript levels were normalized to 18S rRNA levels. Note that the AICDA expression cassette was deleted from all of the lines assayed for GFP transcript levels to avoid effects of nonsense-mediated decay (see Materials and Methods). Data are presented as the mean (± standard error of the mean) of three independent experiments, except for the *hIgλE* 5′ amplicon (2 experiments). Two-tailed unpaired *t*-tests were used to compare the value for each DIVAC element to that of the no DIVAC control. *p<0.05; **p<0.01. (C) Northern blot analysis of *GFP4* transfectants assayed in (B) and two *GFP2* cell lines. Top, hybridization with a GFP probe. Arrows indicate the larger GFP4 and smaller GFP2 mRNA bands; the size difference is as expected from the addition of the hypermutation target sequence to *GFP4*. Bottom, hybridization of the same blot with a control GAPDH probe. Numbers below each lane indicate GFP mRNA levels after normalization to the GAPDH signal expressed relative to the no DIVAC control, which was set to 1. (D) Enhancer function of DIVAC sequences assayed in DT40 cells. Each DIVAC sequence was inserted downstream of a minimal promoter–luciferase gene cassette. Luciferase activity, corrected for transfection efficiency within individual experiments, was normalized to the activity found with the construct containing *hIgλE*, which was arbitrarily set to 1. Data are presented as the mean (± standard error of the mean) of 3–5 independent experiments. Two-tailed unpaired *t*-tests were used to compare data for the *mIgλ* enhancers and shadow enhancers. *p<0.05; **p<0.01.

Consistent with previous studies of the *GFP2* reporter [35],[36] or modifications thereof [37], mRNA levels from *GFP4* were either not significantly or only marginally (up to 2-fold) increased by the presence of chicken or mammalian *DIVAC* fragments compared to the no DIVAC control (Figure 7A–7C). Therefore, as with the GFP2 assay, *DIVAC* elements stimulate mutation in the GFP4 assay by a mechanism that is independent of an increase in GFP transcription.

Given the relatively strong DIVAC function associated with the *mIgλ* shadow enhancers, we wondered whether they also possessed transcriptional enhancer activity. To test this, sequences were cloned downstream of a minimal promoter–luciferase reporter and transfected into the UNG^−/−^AID^R^ recipient cell line used for the GFP4 studies. Both *mIgλE3-1s* and *mIgλE2-4s* were able to stimulate luciferase expression above that of the empty vector (no DIVAC) control, but both exhibited significantly less enhancer activity than their canonical *mIgλ* enhancer counterparts (Figure 7D), despite being stronger *DIVAC* elements. This discordance between transcriptional enhancer activity and DIVAC function further supports the conclusion that *DIVAC* operates by a mechanism distinct from that of stimulating transcription. A very recent study, published while our manuscript was under revision, identified the two *mIgλ* shadow enhancers based on epigenetic criteria and demonstrated that they possess B lineage–specific enhancer activity [51].

## Discussion

Using a highly sensitive, well-controlled assay we provide conclusive evidence that SH is targeted by *Ig* enhancer and *Ig* enhancer-like sequences. The phenomenon is strikingly conserved during vertebrate evolution, as even short mammalian *Igλ* and *IgH* enhancer fragments raised mutation rates more than 20-fold in chicken cells. SH activating sequences, or *DIVAC*, not only physically overlap the *Ig* enhancers but also closely resemble transcriptional enhancers in their mode of action by (i) requiring multiple transcription factor binding sites, (ii) functioning independent of orientation and when positioned either upstream or downstream of the transcription unit, and (iii) increasing activity through the collaboration of multiple enhancer-like regions, each of which depends on transcription factor binding motifs.

The recognition of *Ig* enhancers as SH targeting sequences yields a conceptual framework within which to reevaluate earlier studies. Most notably, the new results vindicate the early transgenic experiments that showed overlap of SH stimulating sequences with the *Igλ*, *IgH* intron, and *Igκ* enhancers [13],[14] and synergistic effects between the *Igκ* intron and *Igκ* 3′ enhancer sequences [13],[15]. The failure of either *Igκ* intron or 3′ enhancer knockouts in mice to abrogate hypermutation [19],[20] is consistent with the contributions of multiple, partially redundant *Igκ* enhancers to *DIVAC* function. Similarly, the failure of a previous study to identify SH targeting function associated with the *Igκ* intron and 3′ enhancers in DT40 cells [38] was likely due to use of a less sensitive assay and the absence of the *Igκ* distal enhancer. In addition, evidence that E-box [37],[52],[53], NFκB [34], MEF2 [34], and PU.1-IRF4 [54],[55] binding sites play a role in the targeting of SH or GCV can be explained by the importance of these sites within the context of *Ig* enhancers and enhancer-like sequences.

The results presented here provide the foundation for models of the *cis*-acting regulatory regions that target SH to a variety of *Ig* loci. The chicken *Igλ* locus is best understood and offers several lessons that might be generally applicable. In *cIgλ*, the enhancer cooperates with an evolutionarily conserved downstream element (*3′Core*) that itself possesses low levels of transcriptional enhancer activity (Figure 7D) but contains functionally important transcription factor binding motifs well known from *Ig* enhancers (Figures S2 and S3). However, it is clear that these two elements depend on additional sequences (e.g., *Con2* and the region between *cIgλE* and *3′Core*) for full DIVAC function (Figures 1 and S6) [35],[36]. The mouse *Igλ* and human and mouse *Igκ* loci offer parallels, with DIVAC function involving the combined action of two or more well-separated enhancer or enhancer-like elements. By analogy with *cIgλ*, it is tempting to think that other surrounding sequences further contribute to the full SH targeting activity of mammalian *Ig* loci. The human *Igλ* enhancer, the human and mouse *IgH* intron enhancers, and a combination of the known *Igκ* enhancers increase SH 20- to 30-fold in our assays, well below the 100-fold stimulation achieved by the full *cIgλ* DIVAC (Figure S6C). Indeed, previous analyses showing that deletion of *mIgHEi* or *hIgHEi* from the endogenous loci did not abolish SH [21],[56] are consistent with the existence of other compensatory targeting elements, a strong candidate for which is the large 3′ regulatory region more than 200 kb downstream of *IgHEi* [57],[58].

The identities of the *trans*-acting factors that bind *Ig* enhancers to stimulate SH are not known, although some candidates have been identified in previous studies and others can be inferred from the binding motifs whose integrity we show is important for DIVAC function. Substantial data support a role for E-box binding factors, including the *E2a*-encoded proteins E12 and E47 [53]. Disruption of *E2a* in DT40 cells reduced the frequency of SH/GCV [59],[60] as did overexpression of the E protein inhibitors Id1 and Id3 [61]. E12 and E47 prefer to bind the CASSTG (S = C or G) subtype of E-box [62], and while mutation of this subtype reduces DIVAC function, mutation of E-boxes predicted to be bound poorly by E12/E47 does also [37]. Existing data leave unresolved the identity of the E-box binding factor(s) that contribute to DIVAC function. Studies in DT40 have also implicated NFκB, PU.1, and IRF4 as *trans* factors relevant for the targeting of SH/GCV [34],[55]. Despite the fact that transcription and hypermutation enhancers make use of overlapping binding motifs and likely an overlapping set of *trans* factors, our data provide a compelling argument that the two processes operate by distinct mechanisms and, in particular, that *DIVAC* does not operate by increasing transcription.

It may not be a coincidence that enhancers, able to exquisitely regulate cell type– and gene-specific expression, have assumed the vital role of targeting SH to the *Ig* loci. The complex structure of *DIVACs*—distinct configurations of a common set of transcription factor binding motifs, with robust activity relying on multiple, and to some extent redundant, sequences—may reflect the formidable task of fine tuning and restricting SH. It might also reflect piecemeal evolution of *DIVAC*, with each *Ig* locus cobbling together an idiosyncratic collection of SH targeting elements. Chromosomal translocations near *DIVACs* likely increase the mutation rate in the neighborhood of the translocation breakpoint, as confirmed for the case of *IgH* to *c-Myc* locus translocations [63]. It is also possible that non-*Ig* genes like *BCL6* that mutate at substantial rates in AID-expressing B cells [10]–[12] do so because of *DIVAC*-like sequences in their neighborhoods. In support of this, a recent computational analysis found that promoter-proximal E-box, C/EBPβ, and YY1 binding motifs (all of which are found in some of the DIVAC elements identified here) were predictive of off-target SH of non-*Ig* genes [64].

Little is known about how gene-specific enhancers and particularly *Ig* enhancers distinguish themselves from other enhancers that may contain the same or similar transcription factor binding sites. Despite this limitation in our understanding of enhancer function, plausible models for how SH is targeted to *Ig* genes can be formulated based on what is known about the interaction of enhancers with the transcription initiation complex (Figure S7). One possibility is that a *DIVAC*-bound factor or a combination of factors actively recruit AID. A not mutually exclusive alternative is that *DIVACs* induce changes in the Pol II transcription initiation or elongation complex, making the transcribed DNA more accessible to AID. This hypothesis might explain why the accumulation of SH events rises rapidly downstream of the transcription start site and then falls off exponentially [3],[65], and might establish a connection between *DIVACs* and stalled transcription [9],[36],[66],[67] or RNA exosome complexes [27]. Interestingly, members of the APOBEC family can induce showers of clustered mutations in breast cancer and yeast cells that are believed to be related to single stranded DNA in the neighborhood of DNA double strand breaks [24],[25], setting a precedent for how a change in DNA conformation can target deaminases to particular regions of the genome.

## Materials And Methods

### Plasmid Construction

The *GFP4* cassette (Figure 1A)—which resembles *GFP2* [35] but contains a 5′ untranslated sequence, the hypermutation target sequence (Figure S1), and, for increased GFP brightness, the *GFP^novo2^* open reading frame [68]—was custom synthesized (Blue Heron Biotechnology) and cloned into the BamHI site of an *AICDA* locus–targeting construct [69], yielding the *GFP4*-containing, *AICDA* locus–targeting construct pAICDA_GFP4. A variant of pAICDA_GFP4, named pAICDA_GFP4D, was made in which the SpeI/NheI sites upstream of GFP4 were deleted and a unique NheI site was introduced downstream of *GFP4*. The cloning of DIVAC sequences into the *GFP4* or *GFP2* targeting vectors is described in Protocol S1.

### Generation of the Recipient UNG^−/−^AID^R/puro^ Cell Clone

An UNG-deficient DT40 clone with both endogenous *AICDA* alleles deleted [32] was reconstituted with AID by the targeted integration of a bicistronic *AICDA*/*gpt* expression cassette into one of the *AICDA* loci [35]. The second *AICDA* locus was subsequently marked by the targeted integration of a puromycin resistance gene driven by the chicken β*-actin* promoter, yielding the recipient UNG^−/−^AID^R/puro^ cell clone for transfections of *GFP4* targeting constructs. The ΨV^−^IgL^−^ clone in which the rearranged *Igλ* locus was replaced by a puromycin resistance cassette [35] was used for transfections of *GFP2* targeting constructs.

### Cell Culture and Flow Cytometry

DT40 cell culture, transfection, drug selection, and the identification of transfectants with targeted integration of *GFP2* constructs were performed as described previously [35]. Transfectants with targeted integration of *GFP4* constructs were also detected by the appearance of puromycin sensitivity. The AID-negative UNG^−/−^AID^−/−^cIgλE↔3′Core clone was derived from the cIgλE↔3′Core transfectant by cre recombinase–mediated removal of the *LoxP*-flanked *AICDA*/*gpt* expression cassette [70].

GFP expression from GFP2 transfectants was assessed by flow cytometry at day 14 after subcloning, as described previously [35],[36], whereas GFP4 transfectants were assessed at day 12 after subcloning. Details of the flow cytometry analysis are provided in Protocol S1.

### Hypermutation Hotspot Sequence Analysis

Genomic DNA was isolated from a subclone of cIgλE↔3′Core after 6 wk of culture and used for the amplification of *GFP4* sequences by PCR using Phusion polymerase (New England Biolabs). The PCR fragments were cloned using the In-Fusion Cloning Kit (Clontech) into the linearized pUC19 provided with the kit and sequenced. Thirty-four sequences covering the first 500 transcribed bases of *GFP4* were aligned to the *GFP4* sequence to detect sequence variation (Figure S1).

### Bioinformatic Analysis

Orthologues of the *Igλ* locus were identified in the turkey, zebra finch, and ground finch genomes using the *W* fragment of *cIgλ* in low stringency blastn BLAST of the reference genome database and Blat genome searches of the respective genome sequences. BLAST and Blat searches were also used to identify the murine *Igλ* shadow enhancers and map them within the murine *Igλ* locus. The bird *Igλ* orthologues were aligned using the ClustalW2 web interface (http://www.ebi.ac.uk/Tools/msa/clustalw2/) to detect sequence contigs conserved during avian evolution. ClustalW2 was also used to create the other sequence alignments shown in Figures S2, S4, and S5. Searches for conserved transcription factor binding sites were performed using the TESS (Transcription Element Search Software) program [71].

### Statistical Analysis

Two-tailed unpaired *t*-tests were used to compare relative *GFP* transcript and luciferase levels in Figures S2D and S2E.

### Gene Expression Analyses

Reverse transcription quantitative PCR analysis was carried out on transfectants containing various DIVAC-GFP4 constructs after the cells were treated with 4-OH tamoxifen and subcloned to delete the AID expression cassette. This avoided potential effects on transcript levels due to nonsense-mediated mRNA decay. The resulting AID-negative cells used for analysis were stably GFP-positive (data not shown). RNA was extracted from 5×10^6^ cells using the RNeasy Mini kit (Qiagen), and the cDNA was prepared from 1 µg of RNA using the iScript cDNA synthesis kit (Bio-Rad). Quantitative PCR was performed using the DyNAmo HS SYBR Green qPCR kit (Thermo Scientific). GFP transcript levels were normalized to 18S rRNA levels. Samples were denatured for 15 min at 95°C, followed by 40 cycles of 30 s at 94°C, 30 s at 60°C, and 30 s at 72°C. The primers used were as follows: GFPup-F 5′-ggaatatactttgccaagaagcgtt-3′, GFP5up-R 5′-accatcgttgccagaaccatt-3′, GFPcds-F 5′-gagcaaagaccccaacgaga-3′, GFPcds-R 5′-gtccatgccgagagtgatcc-3′, 18S-F 5′-taaaggaattgacggaaggg-3′, and 18S-R 5′-tgtcaatcctgtccgtgtc-3′.

RNA for Northern blot analysis was prepared from GFP2 or GFP4 cell lines with the RNeasy kit (Qiagen) or TRIzol reagent (Invitogen). 10 µg of total RNA was run on a gel, transferred to a membrane, and hybridized with a GFP probe. The blot was then stripped and reprobed with a GAPDH probe as a loading control. Using Image Lab software (Bio-Rad), bands were quantitated and normalized to the corresponding GAPDH signal, and values were presented relative to the GFP4 no DIVAC control. The probes were PCR-amplified DNA products made with the corresponding primers: GFPp-F 5′-accatggtgagcaagggcga-3′, GFPp-R 5′-ctaggacttgtacagctcgtccatgc-3′; GAPDHp-F 5′-accagggctgccgtcctctc-3′, GAPDHp-R 5′-ttctccatggtggtgaagac-3′.

### Luciferase Assay

Test sequences were cloned between SalI and BamHI sites downstream of the firefly *Luc2* gene of the minimal promoter containing pGL4.23 vector (Promega). 20 µg of the plasmid was co-transfected into UNG^−/−^AID^R/puro^ cells with 2.5–5.0 µg of pGL4.75 Renilla luciferase control vector (Promega) using the Amaxa Nucleofector kit V (Nucleofector program B-023) (Lonza). The relative activity of firefly luciferase to Renilla luciferase was determined using the Dual-Glo Luciferase Assay System (Promega) according to the manufacturer's protocol.

## Supporting Information

Figure S1 **Introduction of in-frame stop codons by transition mutations within the hypermutation target sequence of *GFP4*.** The top line shows the first 500 base pairs downstream of the *GFP4* transcription start site. The hypermutation target sequence starts with the underlined ATG start codon and ends with the Linker sequence followed by a XbaI site and the *GFP* open reading frame. Hypermutation hotspots (WRCY and its complement RGYW; W = A or T, R = A or G, Y = C or T) are shown in red, with the preferentially mutated base in bold. Mutations in 34 sequences from an UNG-deficient cIgλE↔3′Core subclone after 6 wk of culture are aligned below the *GFP4* sequence, with mutations leading to stop codons in bold. When more than six mutations were seen at a given position, the total number is indicated with a subscript. One 3-bp deletion and a single transversion mutation are shown in blue.
(PDF)

Figure S2 **Alignment of *Igλ* locus sequences from chicken, turkey, zebra finch, and ground finch.** Conserved transcription factor binding motifs, identified as described in Materials and Methods, are indicated. Bases fitting the consensus of the binding motifs are in bold. (A) *Con2* sequences containing a conserved *E-box* and two putative (p) IRF sites, referred to as pIRF-up and pIRF-down to distinguish the upstream and downstream sites. (B) *cIgλE* sequences containing conserved E-box, NFκB, MEF2, and PU.1-IRF4 binding motifs. (C) *3′Core* sequences containing conserved E-box and putative core binding factor (CBF), C/EBP, and PU.1 binding motifs.
(PDF)

Figure S3 **Deletion and mutation analysis of *3′Core*, the second autonomous chicken *Igλ DIVAC* element.** (A) Diagram of the chicken *3′Core* fragment with truncations and deletions indicated below and conserved transcription factor binding motifs depicted as rectangles. The sequences of binding motifs and binding motif mutants are shown on the right. (B) GFP loss of subclones in the presence of full-length, truncated, and internally deleted *3′Core* sequences. GFP4 assay. (C) Diagram of the chicken *3′Core* fragment with binding motif deletions indicated below and conserved transcription factor binding motifs depicted as rectangles. The sequences of binding sites and of binding site mutants are shown on the right. (D) GFP loss of subclones in the presence of binding motif–deleted or binding motif–mutated *3′Core* sequences. The first sample in (B) and (D) depict the same data as one another and as the *3′Core* data of Figure 1D. GFP4 assay.
(PDF)

Figure S4 **Alignment of vertebrate *Igλ* enhancer and mammalian *IgHEi* enhancer sequences.** Conserved transcription factor binding motifs, identified as described in Protocol S1, are indicated. Bases fitting the consensus of the binding motifs are in bold. (A) The upstream and downstream E-box, as well as NFκB, MEF2, and PU.1-IRF4 binding sites are highlighted in the alignment of human, murine and chicken *Igλ* enhancer sequences. (B) Human and murine *IgHEi* enhancer sequences containing conserved YY1 (μE1), E-box (μE2 and μE4), Ets1 (μA), PU.1 (μB), IRF, and Octamer transcription factor binding sites. Also indicated are the well-studied μE5 and μE3 motifs [45],[46],[72], which are not conserved at the sequence level between human and mouse. The mouse μE5 motif binds E proteins such as E47 [72]. Despite poor conservation, the μE3 sites of both mouse and human have been suggested to bind the same factor (core binding factor, CBF) [73].
(PDF)

Figure S5 **Alignment of the human and murine *Igκ* enhancer sequences.** Conserved transcription factor binding motifs, identified as described in Materials and Methods, are indicated. Bases fitting the consensus of the binding motifs are in bold. (A) *IgκEi* sequences containing five conserved E-boxes (κE1, κE2, κE3, and two additional E-boxes in which the CANNTG motif is conserved) and a conserved NFκB binding site. *(B) IgκE3′* sequences containing conserved E-box, NFκB, and PU.1-IRF4 binding sites. (C) *IgκEd* sequences containing conserved E-box, and putative NFκB, PU.1, and IRF binding sites.
(PDF)

Figure S6 **Congruence between the GFP2 and GFP4 assays and analysis of synergy between *cIgλE* and *Con2*.** (A) Diagram of the *Con2-cIgλE* region with truncations of *Con2* indicated below and conserved transcription factor binding motifs depicted as rectangles. The sequences of binding motifs and binding motif mutants are shown on the right. (B) Flow cytometry profiles of representative subclones of primary transfectants carrying either *GFP2* alone (AID^R^) or combined with the *cIgλ* sequence specified above each plot, named according to (A) and Figure 1B. The transfectant named *W* carries the full-length *cIgλ DIVAC* sequence [35]. All transfectants are UNG-proficient, AID-reconstituted. (C) GFP loss in the presence of the indicated DNA elements (GFP2 assay). (D) GFP loss in the presence of the indicated individual DNA elements or composite *Con2*-*cIgλE* elements containing full-length, truncated, or mutated *Con2* sequences (GFP4 assay). The data for *Con2-cIgλE* are the same as in Figure 1D.
(PDF)

Figure S7 **Model for the targeting of SH by *Ig* enhancers.** Recruitment of lymphoid and general transcription factors (some candidate factors are shown [colored ovals]) to multiple *Ig* enhancer and enhancer-like sequences (blue ovals) (top). This leads to the formation of *Ig* enhancer–bound protein complexes that interact by looping with the transcription initiation complex assembled at the *Ig* promoter (middle). It is possible that *Ig* enhancer–bound protein complexes directly or indirectly recruit AID (purple oval) to the transcription initiation complex (middle) to facilitate SH of the *Ig* gene. Alternatively, or in addition, the transcription factors recruited by the *Ig* enhancers might alter parameters of transcription elongation (perhaps increasing Pol II pausing/stalling), thereby increasing the amount of single stranded DNA available for deamination by AID (bottom). While looping involving the enhancers is not depicted in this latter case, it could be occurring at the time of Pol II pausing/stalling.
(PDF)

Protocol S1 **Protocols used for target vector construction and FACS analysis.** Detailed description of the methods used for the construction of the *GFP4* and *GFP2* targeting vectors and for the flow cytometry analysis of GFP fluorescence and calculation of GFP loss.
(DOCX)

## Abbreviations

GCV: *immunoglobulin* gene conversion
SH: somatic hypermutation
