# Supplementary material for: Targeting Of Somatic Hypermutation By immunoglobulin Enhancer And Enhancer-Like Sequences
Source: PLoS Biol. 2014 Apr 1;12(4):e1001831. doi: 10.1371/journal.pbio.1001831 (PMC3972084; doi:10.1371/journal.pbio.1001831)
Supplement: Figure S1 — Introduction of in-frame stop codons by transition mutations within the hypermutation target sequence of GFP4. The top line shows the first 500 base pairs downstream of the GFP4 transcription start site. The hypermutation target sequence starts with the underlined ATG start codon and ends with the Linker sequence followed by a XbaI site and the GFP open reading frame. Hypermutation hotspots (WRCY and its complement RGYW; W = A or T, R = A or G, Y = C or T) are shown in red, with the preferentially mutated base in bold. Mutations in 34 sequences from an UNG-deficient cIgλE↔3′Core subclone after 6 wk of culture are aligned below the GFP4 sequence, with mutations leading to stop codons in bold. When more than six mutations were seen at a given position, the total number is indicated with a subscript. One 3-bp deletion and a single transversion mutation are shown in blue. (PDF) [file pbio.1001831.s001.pdf]

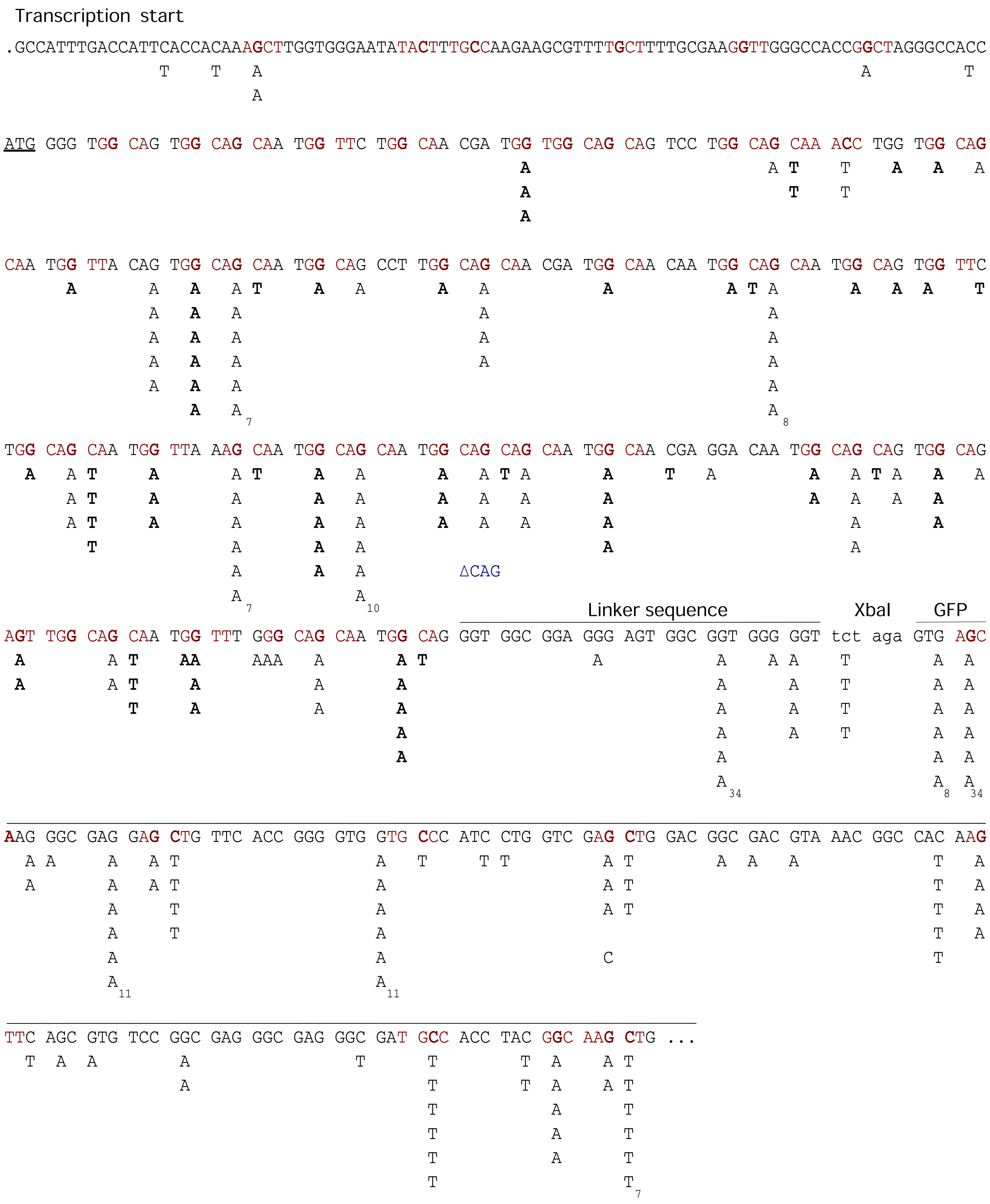
Transcription start
.GCCATTTGACCATTCACCACAAAGCTTGGTGGGAATATACTTTGCCAAGAAGCGTTTTGCTTTTGCGAAGGTTGGGCCACCGGCTAGGGCCACC
ATG GGG TGG CAG TGG CAG CAA TGG TTC TGG CAA CGA TGG TGG CAG CAG TCC TGG CAG CAA ACC TGG TGG CAG
CAA TGG TTA CAG TGG CAG CAA TGG CAG CCT TGG CAG CAA CGA TGG CAA CAA TGG CAG CAA TGG CAG TGG TTC
TGG CAG CAA TGG TTA AAG CAA TGG CAG CAA TGG CAG CAG CAA TGG CAA CGA GGA CAA TGG CAG CAG TGG CAG
ΔCAG
Linker sequence
XbaI
GFP
AGT TGG CAG CAA TGG TTT GGG CAG CAA TGG CAG GGT GGC GGA GGG AGT GGC GGT GGG GGT tct aga GTG AGC
AAG GGC GAG GAG CTG TTC ACC GGG GTG GTG CCC ATC CTG GTC GAG CTG GAC GGC GAC GTA AAC GGC CAC AAG
TTC AGC GTG TCC GGC GAG GGC GAG GGC GAT GCC ACC TAC GGC AAG CTG ...

Figure S1
