# Supplementary material for: Targeting Of Somatic Hypermutation By immunoglobulin Enhancer And Enhancer-Like Sequences
Source: PLoS Biol. 2014 Apr 1;12(4):e1001831. doi: 10.1371/journal.pbio.1001831 (PMC3972084; doi:10.1371/journal.pbio.1001831)
Supplement: Figure S2 — Alignment of Igλ locus sequences from chicken, turkey, zebra finch, and ground finch. Conserved transcription factor binding motifs, identified as described in Materials and Methods, are indicated. Bases fitting the consensus of the binding motifs are in bold. (A) Con2 sequences containing a conserved E-box and two putative (p) IRF sites, referred to as pIRF-up and pIRF-down to distinguish the upstream and downstream sites. (B) cIgλE sequences containing conserved E-box, NFκB, MEF2, and PU.1-IRF4 binding motifs. (C) 3′Core sequences containing conserved E-box and putative core binding factor (CBF), C/EBP, and PU.1 binding motifs. (PDF) [file pbio.1001831.s002.pdf]

A

```
                                                                              E-box
Con2           GGGCTCAGCACGCCTGGCGACGTGGCATCAGCAGAGCAGGCCGCATGG-TACAG-CTCCATCAGCACAGCTGGGGCCACACAAAGAGC
turkey         GGACTCAGCTGGCCTGGCGACGTGGCAGCAACAGAGCAGGCCGCGTGG-TACAG-CTCCATCAGCACAGCTGGGGCCACACAAAGAGC
zebra finch    TGGAGAGGCACG---GGCGGCGTGGCAGAGCCGCAGAGGAGGGGACGGGTCCCCCCTGCCCCAGCCCAGCTGCAGCCACACAAACAGC
ground finch   TGGAGAGGCACG---GGCGGCGTGGCAGAGCCAGAGAGGAGGGGATGGGTCCCGCCTGCCCCCAGGCAGCTGCAGCCGCGCAAGCAGC
                *      **  *   **** *******     *  **  *    *    ** * *    ** *  *     ******  *** * ***  ***

                       pIRF-up pIRF-down
Con2           TGGGTTACTGTGGGCAGCAGG-CTGAAACCCGAAAACAAGAGCTGGGGGCTCAGAATAGCCCCGGGAGCA
turkey         TGGGTTACTGTGGGCAGCAGG-CTGAAACCCGAAAGCAAGAGCTTGGGGCTCAGAATAGCCCTGGGAGCA
zebra finch    C-------TGGAGGCAGCAGGGCTGAAACTCGAAAGCAAGAGCA-GGGTCCCAGAATAGCCCCAG-CCCA
ground finch   CGCGTTCCTGGAGGCAGCAGGGCTGAAACTCGAAAGCAAGAGCA-GGGTCCCAGAATAGCCCCAG-CCCA
                        **   ********* ******* ***** *******   *** * ***********   *    **
```

B

```
                                    E-box                NFκB         MEF2
cIgλE          GTGCAGCACGCTCAGCCCCACCATGCAGCTGTGCGGCCG--GGGCATCCCCAAGCTAAATTTACTTCTCAGTCTCCAATCAGAAACTGAA
turkey         GTGCAGCATGCTCAGCCCCACCGTGCAGCTGTGTGGCCG--GGGCATCCCCGAGCTAAATTTACTCCTCAGTCTCCAATCAGAAACTGAA
zebra finch    TTCCCCCAG--TCAGCCCCACCGTGCAGCTGTGCGGCGGTGGGGCATCCCCTTGCTAAATTTACTCCTCA--CCCACTACAGTCCCC-AA
ground finch   TTTCCCCAG--CCAGCCCCACCGTGCAGCTGTGCAGCAGTGGGGCATCCCCTGGCTAAATTTACTCCTCA--CCCACTACAGCCCCC-AA
                * *  **     ********* *********   ** *   **********  *********** ****   * *    ***     *  **

                                      PU.1    IRF4
cIgλE          GCTGAGGGGCCCACA-CCGGCCAAAAAAAGGAAACGAAACAGTCTCCAGAAAGCACTGACGTGTGAAGCAGAGCGAGCGCCGCGCAAACC
turkey         GCTGGGGGGCCCACG-CTGGCCAAAAAAAGGAAATGAAACAGTCTCCAGAAAGCACTGACGTGTAAAGCAGAGCGAGTGCCGCGCAAACT
zebra finch    TCAAAATTTCGAAAGGGTCCCCCAAAAAAGGAAGTG-AACTGTCTCCAGTGG-TGCTGACGTGTGAAGGCAAGTGAGCGCCACGCGGCCC
ground finch   TCAGAAATTCAAAAGGGCGCCCCAAAAAAGGAAGTGAAACCGTCTCCAGTGGGTGCTGACGTGTGAAGGCAAGAGAGCGCCACGCGGCCG
                *         *   *        ** **********  * *** ********        ********* ***    ** *** *** ***    *
```

C

```
3'Core         TTCAGATGGCCTT-CCCATCTCTCTGCAGCCTCTGCATGGGCTGAACACAAGGTTTAAGTGTTTCTGCCATGTTTTTGGGGCATGTTTGGAGGGGCAGCGTGG----GCCCGGGC
turkey         TTCAGATGGCCTT-CCCGTCCCTCTGCAGCCTCTGCATGGGCTGAGCACATGGCTTAAGCATTTCTGCCATGTTTTTGG--CGTGTTTGAAGGGGCAGTGTGG----ACACG---
zebra finch    AGCCACGGGCCTTTCCCCTTCTTCTGCAGCCACTGCAGGGAAAAACCACCAGCTTTAAACATTTCTGCCATGTTTTGGG--CACTTTTGGCCAGACCCCCCGGGCTGGCTGTGGG
ground finch   AGCCACAGCCCTTTCCCCTTCCTCTGCAGCCACTGCAGGGAAAAACTACCAGGTTTAAACATTTCTGCCATGTTTTGGG--CACTTTTGGCCAGACCCCCCAG-CTGGCTGTGGG
                 *     * **** *** *    ********* ***** **     *  **  *  ****    ************** **  *  *****    *  *       *     *

                                            E-box1                  E-box2   pCBF                      pC/EBP
3'Core         ATACGGGTACTGCCACGTGCCGCCAGCCCCACAGCTGAGCCTG-CACTCTCC----CAGATGTGCTGACCGCAGCCACGGG------GGCAACAGTTTCTCTTGCTAAAAATTG
turkey         ---TGGGTACCGCCACGTGCAGCCAGCGCCACAGCTGAGACTG-CAATCTCC----CAGATGTGCTGACCGCAGCCACAGGCCCGGGGGCAGCAGTTTCTCTTGCTAAAAATTG
zebra finch    CAGGGGGTGCTGATGCACATC-ACAGCCCCCCAGCTGAGCCAGGCACACTCACAGACAGCTGTTTTG-CCGCAGCCCCGCAGCTGGAAA-CAG------CCCTTGCTAAAATTAC
ground finch   CAGGGG--TCTGATGCACATC-ACAGCCCCCCAGCTGAGCCAGGCACCCTCCCAGGCAGCTGTTTTGACCGCAGCCCCGCAGGTTGGAAACAG------CCCTTGCTAAAATTAC
                   **   * *   *       **** ** ******** * * **  ***      *** ***  ** ******** *              **         ********** *

                                                                      E-box3      pPU.1
3'Core         ----------------TAGCCGGGAAGAAAACACGTGGCAACTTCGGCCAAACAGCAGCTGGAGGACAGGAATAGCCGTGGCCACGGCACGCTCTGCTTCCTCGGCACAAACATT
turkey         ----------------TGGCCGGGCCGAAAACATGAGGCCACTTCGGCCAAACAGCAGCTGGAGGACAGGAATAGCTGTGGCCATGGCATGCTCTGCTTCCTCGGCACAAACATT
zebra finch    AGCCCGCCAAAACCAAGAGCCCG-CCAAAACCAAGAGCCCGCTTGG-CCAAACATCAGCTGGCAGCCAGGAATAGCCCCGGCCTCGCCACGCTCCGCTGCCTTGGCACAAACCTT
ground finch   AGCCCGCCAAAAGCGAGAGCCCG-CCAAAAGCGAGAGCCCGCCTGGGCCAAACATCAGCTGGGAGCCAGGAATAGCCCCGGCCCCG-CTCGCTCCGCTGCCCTGGCACAAAGTGC
                                *** *    *** *  * * *   * * * ******* *******  * **********   ****  * *  **** *** **  ********

3'Core         CCAGTACGTGGCACCACGAGCGCCGCTGCCCGGCACAGCAGCAAGCAGAGCCAGGAGCAGGAAATGCTG-ATTTGGGCCCCATTTTGGCCATGGCTGAGAGA
turkey         CCAGCACGTGGCACCATGAGTGCCACTGCCCGGCACAGCAGGAAGCAGAGCCAGGAGCAGGAAATGCTG-ATTTGGGCCCCATTTTGGTAATGGCTCAGAGA
zebra finch    CCCTCACATGGCACC-CGAGCACCGCTGCCTGGCCGTGCCGCGGGCAGG--CAAAAGCGGGAAAAGCTGGGGTTTGGCCCCAGTTCAGCAGTGGCTCATGGT
ground finch   CCTGCACGTGGCACC-CGGGCACCCCTGCCGGGCCGTGCCAGGGCAGGG--CAAGGGCGGGAAAATCCGGGGTTTGGCCCCAACTCAGCAACGGCTCATGGA
               **   *******      * *  ** ***** ***    **          *    **    ** *****   * * . ** ****** * *   *    **** *  *
```

Figure S2
