# Supplementary figures and images for: Targeting Of Somatic Hypermutation By immunoglobulin Enhancer And Enhancer-Like Sequences
Source: PLoS Biol. 2014 Apr 1;12(4):e1001831. doi: 10.1371/journal.pbio.1001831 (PMC3972084; doi:10.1371/journal.pbio.1001831)

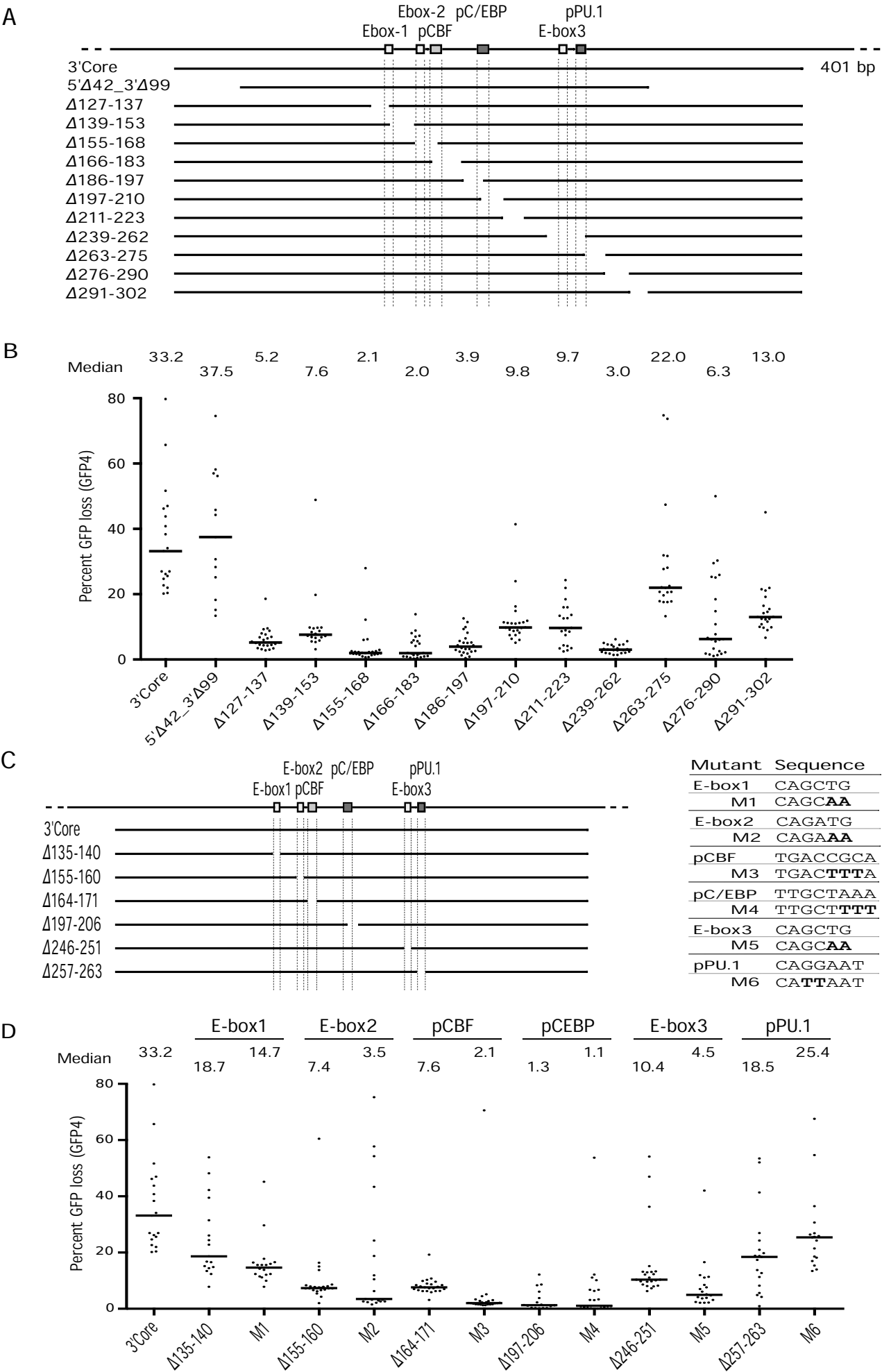

Figure S3

Supplement: Figure S3 — Deletion and mutation analysis of 3′Core, the second autonomous chicken Igλ DIVAC element. (A) Diagram of the chicken 3′Core fragment with truncations and deletions indicated below and conserved transcription factor binding motifs depicted as rectangles. The sequences of binding motifs and binding motif mutants are shown on the right. (B) GFP loss of subclones in the presence of full-length, truncated, and internally deleted 3′Core sequences. GFP4 assay. (C) Diagram of the chicken 3′Core fragment with binding motif deletions indicated below and conserved transcription factor binding motifs depicted as rectangles. The sequences of binding sites and of binding site mutants are shown on the right. (D) GFP loss of subclones in the presence of binding motif–deleted or binding motif–mutated 3′Core sequences. The first sample in (B) and (D) depict the same data as one another and as the 3′Core data of Figure 1D. GFP4 assay. (PDF) [file pbio.1001831.s003.pdf]

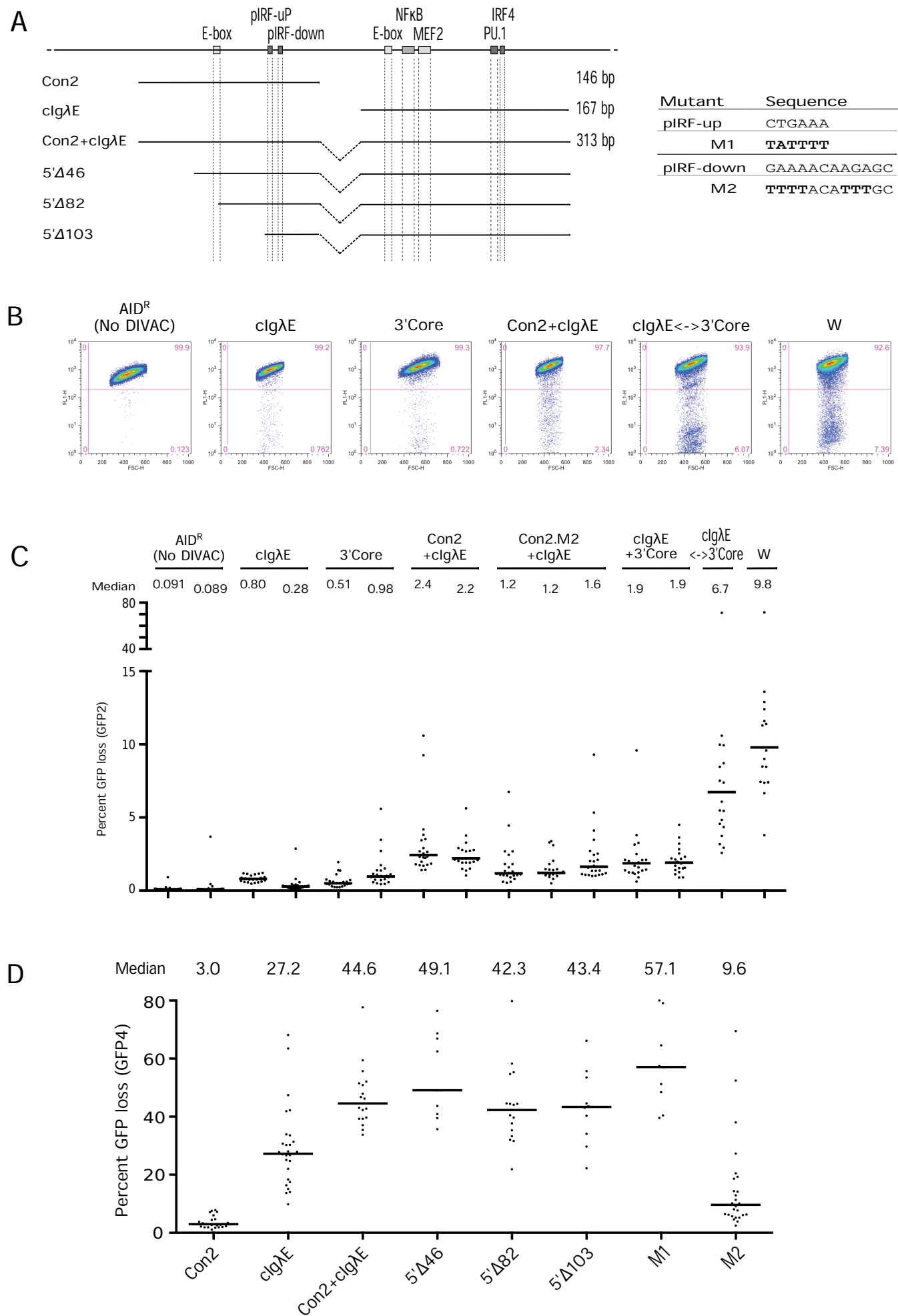

Figure S6

Supplement: Figure S6 — Congruence between the GFP2 and GFP4 assays and analysis of synergy between cIgλE and Con2. (A) Diagram of the Con2-cIgλE region with truncations of Con2 indicated below and conserved transcription factor binding motifs depicted as rectangles. The sequences of binding motifs and binding motif mutants are shown on the right. (B) Flow cytometry profiles of representative subclones of primary transfectants carrying either GFP2 alone (AIDR) or combined with the cIgλ sequence specified above each plot, named according to (A) and Figure 1B. The transfectant named W carries the full-length cIgλ DIVAC sequence [35]. All transfectants are UNG-proficient, AID-reconstituted. (C) GFP loss in the presence of the indicated DNA elements (GFP2 assay). (D) GFP loss in the presence of the indicated individual DNA elements or composite Con2-cIgλE elements containing full-length, truncated, or mutated Con2 sequences (GFP4 assay). The data for Con2-cIgλE are the same as in Figure 1D. (PDF) [file pbio.1001831.s006.pdf]

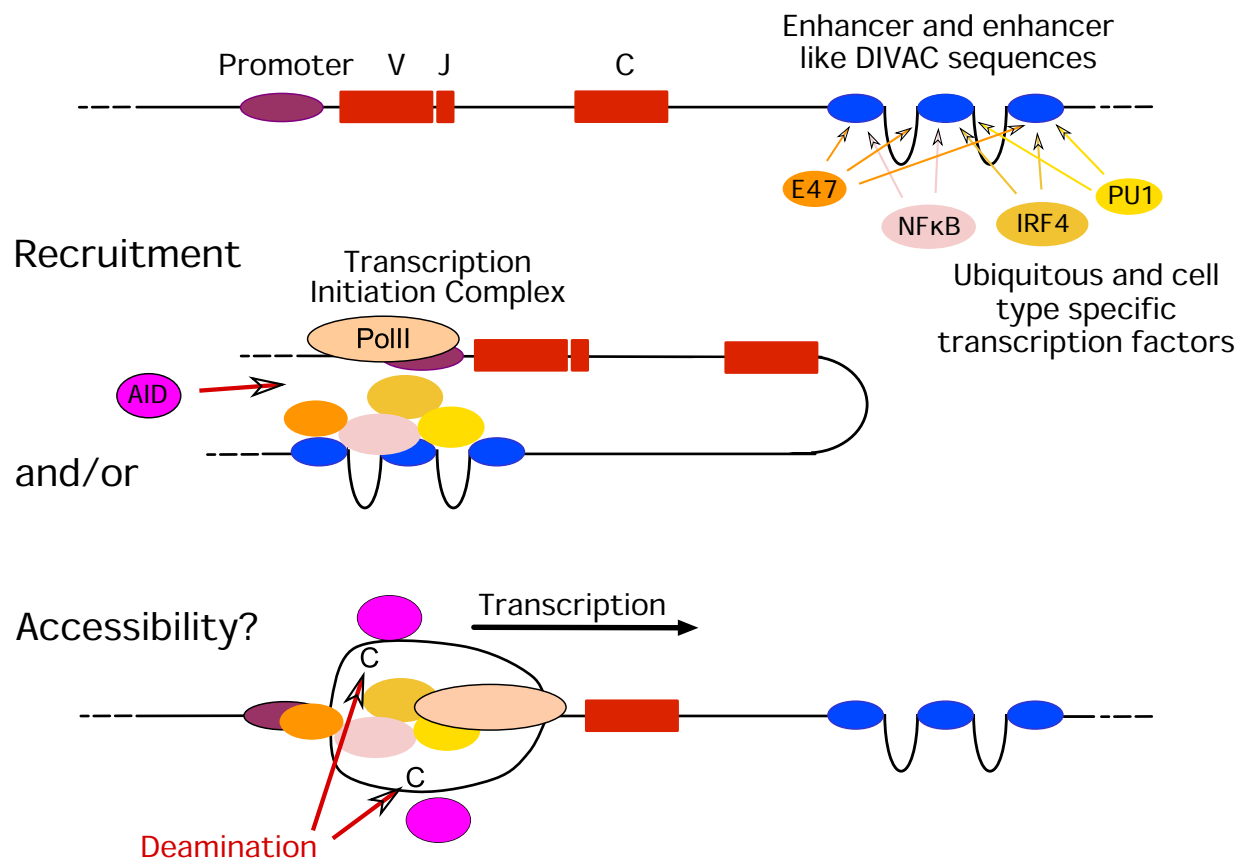

Figure S7

Supplement: Figure S7 — Model for the targeting of SH by Ig enhancers. Recruitment of lymphoid and general transcription factors (some candidate factors are shown [colored ovals]) to multiple Ig enhancer and enhancer-like sequences (blue ovals) (top). This leads to the formation of Ig enhancer–bound protein complexes that interact by looping with the transcription initiation complex assembled at the Ig promoter (middle). It is possible that Ig enhancer–bound protein complexes directly or indirectly recruit AID (purple oval) to the transcription initiation complex (middle) to facilitate SH of the Ig gene. Alternatively, or in addition, the transcription factors recruited by the Ig enhancers might alter parameters of transcription elongation (perhaps increasing Pol II pausing/stalling), thereby increasing the amount of single stranded DNA available for deamination by AID (bottom). While looping involving the enhancers is not depicted in this latter case, it could be occurring at the time of Pol II pausing/stalling. (PDF) [file pbio.1001831.s007.pdf]
