# Supplementary material for: Targeting Of Somatic Hypermutation By immunoglobulin Enhancer And Enhancer-Like Sequences
Source: PLoS Biol. 2014 Apr 1;12(4):e1001831. doi: 10.1371/journal.pbio.1001831 (PMC3972084; doi:10.1371/journal.pbio.1001831)
Supplement: Figure S5 — Alignment of the human and murine Igκ enhancer sequences. Conserved transcription factor binding motifs, identified as described in Materials and Methods, are indicated. Bases fitting the consensus of the binding motifs are in bold. (A) IgκEi sequences containing five conserved E-boxes (κE1, κE2, κE3, and two additional E-boxes in which the CANNTG motif is conserved) and a conserved NFκB binding site. (B) IgκE3′ sequences containing conserved E-box, NFκB, and PU.1-IRF4 binding sites. (C) IgκEd sequences containing conserved E-box, and putative NFκB, PU.1, and IRF binding sites. (PDF) [file pbio.1001831.s005.pdf]

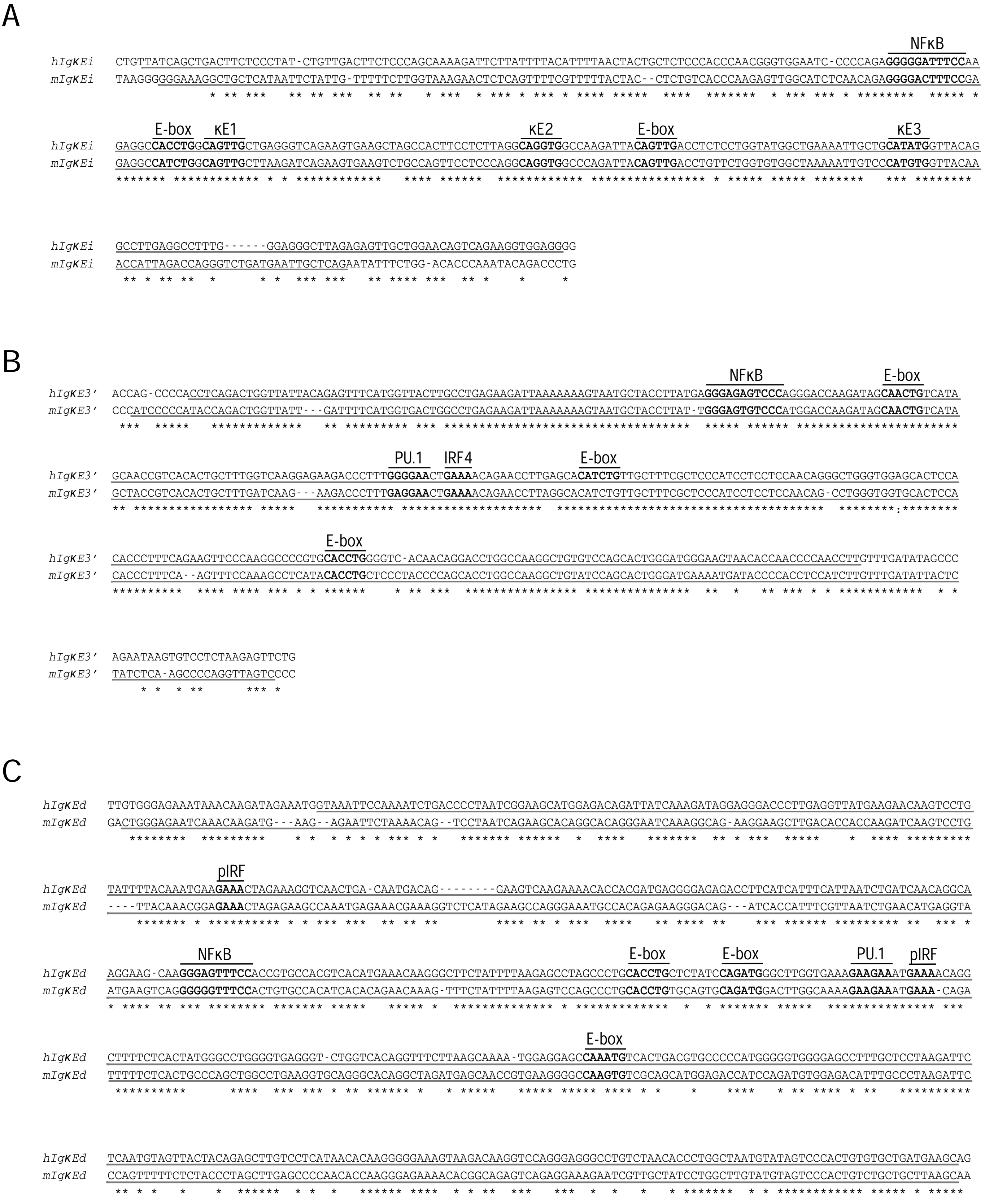
A
NFκB
E-box
κE1
κE2
E-box
κE3
hIgκEi
mIgκEi
B
NFκB
E-box
PU.1
IRF4
E-box
E-box
hIgκE3'
mIgκE3'
C
pIRF
NFκB
E-box
E-box
PU.1
pIRF
E-box
hIgκEd
mIgκEd

Figure S5
