# Supplementary material for: Targeting Of Somatic Hypermutation By immunoglobulin Enhancer And Enhancer-Like Sequences
Source: PLoS Biol. 2014 Apr 1;12(4):e1001831. doi: 10.1371/journal.pbio.1001831 (PMC3972084; doi:10.1371/journal.pbio.1001831)
Supplement: Protocol S1 — Protocols used for target vector construction and FACS analysis. Detailed description of the methods used for the construction of the GFP4 and GFP2 targeting vectors and for the flow cytometry analysis of GFP fluorescence and calculation of GFP loss. (DOCX) [file pbio.1001831.s008.docx]

# Detailed Protocols

## Buerstedde et al., Targeting of somatic hypermutation by *immunoglobulin* enhancer and enhancer-like sequences

### Cloning of *DIVAC* sequences into *GFP4* and *GFP2* targeting vectors

Potential *DIVAC* sequences were amplified by PCR using Phusion Polymerase (New England Biolabs) and either the *W* fragment of the *cIgλ* locus, DT40 genomic DNA, or genomic DNA from human and murine cell lines as templates. Sequences to be tested in the GFP4 assay were inserted into SpeI/NheI sites of the pAICDA_GFP4 vector, or in the case of *hIgλEDown* into the NheI site of pAICDA_GFP4D. Sequences to be tested in the GFP2 assay were inserted into the SpeI/NheI sites of the *GFP2* containing, *cIgλ* locus targeting construct pIgLGFP2 [[1](#_ENREF_1)]. Directional cloning was accomplished using the highly efficient In-Fusion Cloning Kit (Clontech), and all sequences were inserted in the forward orientation except for *cIgλER* and *3'CoreR* as indicated in Figure 1B. Sequences containing internal deletions were generated by adding a 15 bp overlap to PCR amplifications of the upstream and downstream sequences flanking the deletion, followed by simultaneous insertion of the PCR products into the *GFP4* or *GFP2* targeting constructs using the In-Fusion Cloning Kit. Sequences containing binding site mutations were similarly produced by incorporating the mutations and 15 base pair overlaps into PCR amplifications of the upstream and downstream flanking sequences followed by simultaneous insertion of the PCR products into the targeting constructs using the In-Fusion Cloning Kit. Combinations of *DIVACs* were produced by adding 15 bp overlaps to PCR products containing the individual *DIVACs* to be combined and simultaneous insertion using the In-Fusion Cloning Kit. The sequences of all inserts were confirmed by sequence analysis before transfection. The *GFP4* and *GFP2* targeting constructs were linearized before transfection by XhoI and NotI respectively.

### FACS analysis

Certain GFP2 transfectants, such as those with a weak *DIVAC* element, yield very low percentages of GFP loss, making detection and quantitation of these small populations more difficult. To overcome this, 50,000 live events were collected for each GFP2 subclone analyzed. The same gates were used for all transfectants. As AID^R^ subclones expressed slightly lower GFP levels than the subclones of other transfectants (Figure S6B), this analysis likely overestimates the amount of GFP loss in the absence of DIVAC compared to a previous analysis in which gates were adjusted for different transfectants [[1](#_ENREF_1)].

GFP4 transfectants typically yield substantial levels of GFP loss and hence 5000 live events were collected for GFP4 subclones, except for subclones of the AID-negative UNG^-/-^AID^-/-^cIgλE<->3'Core clone for which 50,000 live events were collected. Since the few negative events in UNG^-/-^AID^-/-^cIgλE<->3'Core subclones often localized close to the upper boundary of the negative gate, the analysis of Figure 1C and 1D likely overestimated the very low levels of GFP loss that occur in AID-negative GFP4 transfectants. Fluorescence levels of GFP-positive GFP4 transfectants was substantially lower than that of GFP2 transfectants, likely reflecting either mRNA or protein instability or impaired GFP folding due to the added *Hypermutation Target Sequence* (Figure 1B and S6B). However, GFP positive and negative cells could be easily distinguished because GFP loss in GFP4 transfectants was usually complete, as expected from the introduction of in-frame stop codons in the *Hypermutation Target Sequence*. Subclones with greater than 80% GFP loss were excluded from the analysis because of the possibility that they derived from a GFP-negative cell at the time of subcloning.

## References

1. Blagodatski A, Batrak V, Schmidl S, Schoetz U, Caldwell RB, et al. (2009) A cis-acting diversification activator both necessary and sufficient for AID-mediated hypermutation. PLoS Genet 5: e1000332.
